# Empirical evidence for robust personality-gaming disorder associations from a large-scale international investigation applying the APA and WHO frameworks

**DOI:** 10.1371/journal.pone.0261380

**Published:** 2021-12-22

**Authors:** Christian Montag, Christopher Kannen, Bruno Schivinski, Halley M. Pontes

**Affiliations:** 1 Department of Molecular Psychology, Institute of Psychology and Education, Ulm University, Ulm, Germany; 2 School of Media and Communication, RMIT University, Melbourne, VIC, Australia; 3 Department of Organizational Psychology, Birkbeck, University of London, London, United Kingdom; Yale University, UNITED STATES

## Abstract

Disordered gaming has gained increased medical attention and was recently included in the eleventh *International Classification of Diseases* (ICD-11) by the World Health Organization (WHO) after its earlier inclusion in the *Diagnostic and Statistical Manual of Mental Disorders* (fifth revision) (DSM-5) as an emerging disorder by the American Psychiatric Association (APA). Although many studies have investigated associations between personality and disordered gaming, no previous research compared the differential associations between personality and disordered gaming with time spent gaming. Due to the novelty of the WHO diagnostic framework for disordered gaming, previous research focused mainly on the associations between personality and disordered gaming in relation to the APA framework. Beyond that, these studies are generally limited by small sample sizes and/or the lack of cross-cultural emphasis due to single-country sampling. To address these limitations, the present study aimed to investigate the associations between personality and gaming behavior in a large and culturally heterogeneous sample (N = 50,925) of individuals from 150 countries. The results obtained suggested that low conscientiousness and high neuroticism were robustly associated with disordered gaming across both the APA and WHO frameworks. Interestingly, personality associations with weekly time spent gaming were smaller. The findings of the present study suggest that personality is of higher importance to predict disordered gaming compared to weekly time spent gaming.

## 1. Introduction

Disordered gaming has been recently recognized as an official addictive disorder without the use of substances alongside Gambling Disorder [1]. Currently, this condition can be examined both in research and clinical milieus using the American Psychiatric Association (APA) framework [2] and/or the World Health Organization (WHO) framework [3]. The status of disordered gaming as a mental health condition has gained prominence in terms of its medical significance since the latest fifth revision of the *Diagnostic and Statistical Manual of Mental Disorders* (DSM-5; American Psychiatric Association, 2013) when the term ‘Internet Gaming Disorder’ (IGD) appeared as a tentative mental health disorder within Section III, as an emerging disorder requiring additional research.

According to the APA framework, IGD reflects the experience of the following nine diagnostic criteria within a 12-month timeframe: (1) preoccupation with gaming; (2) withdrawal symptoms when gaming is not possible; (3) tolerance, leading to the need to spend higher amounts of time gaming; (4) unsuccessful attempts to control the behavior; (5) loss of interest in hobbies as a result of, and with the exception of gaming; (6) continued excessive gaming behavior despite resulting psychosocial problems; (7) deception of significant others in relation to the amount of gaming; (8) gaming as a way to escape or relieve negative mood; and (9) jeopardizing or losing social relationships, academic or professional opportunities due to gaming [2].

After intense scholarly debates regarding the medical relevance of disordered gaming [4, 5], the WHO framework defined ‘Gaming Disorder’ (GD) and included it as a *bona fide* mental health disorder in the eleventh revision of the *International Classification of Diseases* (ICD-11), which specifies that GD results from the experience of three core symptoms, also usually observed within a 12-month timeframe, reflecting (1) significant problems in controlling one’s own gaming behavior, (2) upholding gaming despite negative effects on one’s own life, and (3) heightened importance given to gaming to the detriment of other important daily life activities [3]. The WHO advises that this condition should only be diagnosed when the behavior leads to significant impairments in everyday life (e.g., academic, professional, inter-personal, etc.). Although the two diagnostic frameworks map on the same construct, a recent study [6] supported the validity of the new WHO framework, but also pointed out several problems with the APA framework for disordered gaming.

Notwithstanding this, in terms of the epidemiological evidence underpinning disordered gaming, a recent meta-analysis reported a worldwide prevalence of 1.96% [7]. However, the epidemiological evidence used in this meta-analytical study only assessed disordered gaming based on the APA framework and not the most recent WHO framework.

With regards to potential etiological factors pertaining to disordered gaming, the Interaction of Person-Affect-Cognition-Execution (I-PACE) model [8] provides a prominent theory-driven framework that sheds light on the development of addictive behaviors such as disordered gaming. Accordingly, the acronym I-PACE alludes to the interaction of person, affect, cognition, and execution variables that may enhance individual acquisition and development of addictive behaviors. Of interest to the present study, a systematic review [9] provided evidence that the individual’s personality is a relevant variable that is capable of providing further understanding regarding why a person or groups might be more likely to develop disordered gaming symptoms. Indeed, previous research highlighted the role that personality may play in developing risky behaviors in relation to addictive processes. Personality traits are key risk factors in the development and maintenance of Internet Use Disorders such as disordered gaming, particularly in relation to specific traits such as neuroticism [10, 11], impulsivity [12], and sensation seeking [13] as shown in the literature.

In terms of the classic Big Five personality trait model [14], the literature has further highlighted neuroticism as a critical vulnerability factor for augmented disordered gaming tendencies alongside higher impulsivity and aggressiveness tendencies [9]. Furthermore, existing empirical evidence also pointed to high introversion scores as relevant predictors of disordered gaming (beyond neuroticism [15]), with the extraversion/introversion link not being observed by others [16].

Given that the Big Five personality traits arguably pertain to the most prominent theoretical framework to study individual differences within psychological and social sciences, it is paramount to further investigate the associations between personality traits and disordered gaming and the robustness of these links within large-scale studies. To help advance this important line of research, the present study was conducted in a large sample with a heterogenous cultural background, while also considering both the APA and WHO frameworks to assess the consistency of the findings in relation to these two diagnostic instruments.

Since previous recent research found important differences within the two most widely used diagnostic frameworks for disordered gaming, particularly in relation to its epidemiology [17, 18], it is key to further explore the ways in which individual differences may be differentially associated with disordered gaming across these two frameworks. Moreover, given a recent discussion on the relationships between time spent gaming and personality traits [19], the current study further addresses and explores this issue as lower conscientiousness, lower extraversion, and lower agreeableness were all associated with greater time spent gaming [19]. There are several important reasons underpinning the need to also investigate time spent gaming in the present study. As can be seen in the diagnostic guidelines for GD within the WHO framework, (excessive) time spent gaming does not represent a symptom to be considered in its diagnosis. Disordered gaming is a behavior in which large amounts of time spent gaming is typically and logically observed. However, not everyone spending large amounts of time on gaming will show disordered gaming patterns (e.g., professional gamers earning a living through gaming would not necessarily classify as disordered gamers upon excessive engagement). Thus, the intricate relationship between time spent gaming in the context of disordered gaming merits further investigation.

Based on previous findings [20], we expect neuroticism to be positively associated with disordered gaming. Additionally, in line with a recent meta-analysis on other (mobile) technological use disorders [21], a negative association between disordered gaming and conscientiousness is also expected. Furthermore, based on the exploratory nature of the present investigation, we expect that differential relationship patterns will emerge between disordered gaming and time spent gaming (a recent work [19] suggests only very small associations). Finally, as recent studies [17, 18] indicate that both IGD (i.e., APA framework) and GD (i.e., WHO framework) constructs are highly correlated, and as both measure disordered gaming behaviors, it is also expected that emerging personality correlates will be largely equivalent across the APA and WHO frameworks.

## 2. Methods

### 2.1. Background on data collection

The current study was part of a large global campaign to foster responsible and healthy gaming behaviors that was widely disseminated through an online platform called www.do-i-play-too-much.com in partnership with the Electronic Sports League (ESL). The role of ESL was to provide visibility to the online platform among gamers without having any direct or indirect input on the present study. Thus, ESL publicized our online platform in the context of the ongoing “Smart Gaming” campaign (https://about.eslgaming.com/portfolio/smart-gaming) via their website and social media channels. It is worth noting that ESL did not fund the present project (see also conflict of interest section). Beyond this, participation in the study was incentivized by providing each participant with an anonymized and bespoke feedback on their tendencies towards disordered gaming and personality in comparison to participants that had completed our survey at that time. This procedure was also adopted to encourage participants to fill in the survey in a meaningful way as not doing so would lead to biased feedback about their own behaviors.

Data was collected online via the aforementioned platform, which was also heavily promoted via institutional press releases from the universities of the involved scientists, and featured on several websites, specialized gaming forums, online magazines, news platforms, radio broadcasts, among other sources. As a result, a total of 68,132 individuals participated in the present study from May to December 2019. Participants filled out the online survey containing several psychometric tests related to basic demographic, gaming, and personality variables. For readability purposes, detailed information on the number of participants from different countries is provided as S1 Data (for the cleaned data set, see below).

Although the online platform (i.e., Smart Gaming campaign) is still running as this is an ongoing project, for the current study we decided to analyze a particular temporal subset of the data set encompassing only pre-COVID data to minimize potential confounding effects due to the links between the effects of the pandemic and increased mental health disturbances, such as GD [22]. The study has received ethical approval by the research team’s University Ethics Committee (PONTES 2018/95, Nottingham Trent University).

### 2.2. Data cleaning strategy

Individuals were allowed to partake in the study if they were aged at least 12 years old. Respondents aged between 12–15 years had to provide an additional authorization by their legal guardians. Participants not meeting the specified age range and/or not complying with the electronic consent requirements of the study were not included.

We further filtered out those who reported to be over 80 years old and not proficient in English as the survey was devised in English language. To help improving the quality of the data, a sham item was included in the survey asking participants if they had played the fictional game *Semeron* (yes/no). Respondents stating “yes” to this item were not included in the formal statistical analyses. We also excluded those who reported not playing any video games in the last 12 months and those providing implausible time spent gaming (i.e., above 112 self-reported hours of gaming time per week, assuming an average of 7 hours of sleep each night; and no more than 48 hours of self-reported gaming activities over the weekend alone). The final filtering procedure included using the R [23] package careless (version 1.2.1) [24] and the *longstring()* function to detect carelessness and insufficient effort when completing the survey.

After implementing the aforementioned data cleaning steps, the data set (which contained several other scales and constructs relevant to the overall objective of the project) was subset to include participants that completed the surveys on Big Five personality traits and disordered gaming behavior according to both the APA and WHO frameworks (further detailed in section 2.3. Measures). The final sample for the current study consisted of 50,925 participants (mean_age_ = 24.85 years; SD = 7.38) from a total of 150 countries, with about 90.3% (n = 45,996) reporting being male. Finally, the data presented with no missing values as participants were asked to fill in the survey completely in order to receive meaningful feedback on their gaming behaviors.

### 2.3. Measures

All eligible participants provided information on sociodemographic variables, personality, disordered gaming, and weekly time spent gaming. The Big Five personality traits were assessed with the Big Five Inventory (BFI-44) [25], which is a psychometric test that assesses the five personality dimensions of openness (α = .74, McDonald’s Ω = .80), conscientiousness (α = .78, Ω = .81), extraversion (α = .82, Ω = .86), agreeableness (α = .72, Ω = .77), and neuroticism (α = .83, Ω = .86), with higher scores indicating higher tendencies toward each personality trait. The internal consistency of the scales was adequate and above the recommended threshold of .70 [26]. The BFI-44 was answered with a five Likert scale ranging from 1 = “disagree strongly” to 5 = “agree strongly”.

Further psychometric assessment included examining the APA framework for disordered gaming with the Internet Gaming Disorder Scale–Short-Form (IGDS9-SF) [27] and the WHO framework with the Gaming Disorder Test (GDT) [18]. The IGDS9-SF includes nine items while the GDT has four items, with higher scores on the two tests suggesting greater levels of disordered gaming tendencies across both frameworks. Both scales are answered with a five Likert scale ranging from 1 = “never” to 5 = “very often”. With respect to the WHO framework answering all four items of the GDT with 4 = “often” or 5 = “very often” would result in our work in the category “disordered gaming”. With respect to the APA framework answering five out of nine items of the IGDS9-SF with 4 = “often” or 5 = “very often” would result in our work in the category “disordered gaming”. Since the present study had a cross-cultural emphasis, these two disordered gaming psychometric tests were chosen due to their robust cross-cultural suitability and extensive evidence within different cultural settings as evidenced by a number of studies [17, 28]. In the present sample, both the IGDS9-SF (α = .83, Ω = .85) and the GDT (α = .77, Ω = .79) had adequate levels of internal consistency.

### 2.4. Statistical analysis

All statistical analyses were performed using IBM SPSS and R programming language for statistical computing and graphics version 4.0.4 "Lost Library Book" [23]. Regarding the R analysis: To investigate the role of gender-based differences both frequentist and Bayesian methods were adopted by computing Welch’s *t*-test statistics using the *BayesFactor* package (version 0.912–4.2) [29]. Further, correlational analysis between the main variables of the study was conducted with the *psych* package (version 2.1.3) [30] using Holm *p*-value adjustments for multiple simultaneous testing. Finally, a multiple linear regression model was estimated with base R to also investigate the role of personality traits in predicting disordered gaming across both the APA and WHO frameworks. The aggregated data used in the present study can be found here: https://osf.io/ntyhr/.

## 3. Results

### 3.1. Main descriptive statistics and gender-based differences findings

A full summary of the main descriptive and gender-based findings is presented in Table 1. While gender had a statistically significant effect on weekly time spent gaming, openness, conscientiousness, extraversion, agreeableness, and neuroticism, no gender effects were observed on disordered gaming according to both APA and WHO frameworks (the influence of gender on IGD was *p* = .04, but this would not hold for multiple testing).

**Table 1:** Descriptive statistics and gender-based analysis of relevant variables.

*Variables*	Overall Sample mean (SD)	Male Sample (mean, SD)	Female Sample (mean, SD)	*t*-statistic (df)	*p*	Bayes Factor (BF_10_)	Cohen’s d
Gaming Disorder	9.05 (3.26)	9.05 (3.25)	9.01 (3.35)	-0.84 (5960.1)	.40	-3.73	-.01
Internet Gaming Disorder	18.25 (6.25)	18.23 (6.22)	18.43 (6.52)	2.03 (5931.2)	.04	-2.03	.03
Weekly Gaming Time (hours)	24.43 (16.94)	24.66 (16.89)	22.21 (17.23)	-9.52 (5988.7)	< .001	41.19	-.14
Openness	3.63 (0.58)	3.62 (0.58)	3.71 (0.60)	10.58 (5972.7)	< .001	51.74	.16
Conscientiousness	3.21 (0.66)	3.21 (0.66)	3.15 (0.71)	-6.13 (5880.1)	< .001	14.70	-.09
Extraversion	2.75 (0.78)	2.77 (0.78)	2.63 (0.79)	-11.84 (6009.3)	< .001	65.88	-.17
Agreeableness	3.52 (0.62)	3.53 (0.61)	3.47 (0.67)	-6.07 (5837.2)	< .001	14.32	-.09
Neuroticism	2.94 (0.83)	2.88 (0.82)	3.44 (0.82)	45.45 (6011.8)	< .001	> 100	.68

Note: *t*-statistic = Welch’s *t*-test statistic; df = degrees of freedom; SD = standard deviation.

Gaming Disorder as measured by the Gaming Disorder Test (GDT); Internet Gaming Disorder as measured by the Internet Gaming Disorder Scale–Short-Form (IGDS9-SF).

Overall Sample: N = 50,925, Male Sample: n = 45,996, Female Sample: n = 4,929.

### 3.2. Associations between personality and disordered gaming

The main associations between personality and gaming variables (and other relevant variables) are presented in Table 2. Substantial associations were found between high neuroticism and disordered gaming across both the APA and the WHO frameworks. Additionally, robust associations were also found between low conscientiousness with disordered gaming across both APA and WHO frameworks.

**Table 2:** Associations between personality traits, disordered gaming, time spent gaming, age, and gender.

	Openness	Conscientiousness	Extraversion	Agreeableness	Neuroticism
Gaming Disorder	-.11	-.37	-.16	-.19	.27
Internet Gaming Disorder	-.12	-.33	-.19	-.21	.33
Weekly Time Spent Gaming	-.10	-.12	-.10	-.09	.07
Age	.11	.17	-.01	.09	-.04
Gender	.05	-.03	-.05	-.03	.20

**Note**: All correlation coefficients remained statistically significant at *p* < .001 after applying Holm *p*-value correction.

Gaming Disorder as measured by the Gaming Disorder Test (GDT); Internet Gaming Disorder as measured by the Internet Gaming Disorder Scale–Short-Form (IGDS9-SF).

As predicted, associations between all five personality traits with weekly time spent gaming were significantly less pronounced compared to disordered gaming (see Table 2). For the sake of completeness and interested readers, we present correlations regarding personality traits and disordered gaming for each country representing a minimum of 1% of the complete sample (see S1 Data).

### 3.3. The role of personality traits in predicting disordered gaming

In terms of the predictive model using multiple linear regression to ascertain how personality traits may predict disordered gaming severity, the two models were statistically significant and explained a highly comparable amount of total variance in disordered gaming on both the APA and WHO frameworks (i.e., Adjusted R^2^_APA framework_ = .226; Adjusted *R*^2^_WHO framework_ = .223). The findings further indicated that the most informative personality traits were conscientiousness (β_APA framework_ = -.201, *t* = -46.284, *p* < .001; β_WHO framework_ = -.275, *t* = -63.04, *p* < .001) and neuroticism (β_APA framework_ = .214, *t* = 46.948, *p* < .001; β_WHO framework_ = .135, *t* = 29.502, *p* < .001) as they exerted the most predictive influence on disordered gaming. Please note that in these models also age, gender and time spent gaming were included (see Table 3).

**Table 3:** Multiple linear regression of the relationship between disordered gaming and key individual differences predictors.

	Parameter estimates						
*Predictors*	*B*	SE	β	*t*-statistic	*p*-value	95% Confidence Interval	
**Outcome: Gaming Disorder**							
(Intercept)	11.831	.160	-	74.034	< .001	11.517	12.144
Age	-.005	.002	-.011	-2.674	.008	-.008	-.001
Gender	.332	.044	.030	7.535	< .001	.246	.419
Weekly time spent gaming	.046	.001	.240	60.440	< .001	.045	.048
Openness	-.051	.023	-.009	-2.142	.032	-.096	-.004
Conscientiousness	-1.347	.021	-.275	-63.041	< .001	-1.389	-1.305
Extraversion	-.062	.018	-.015	-3.395	.001	-.098	-.026
Agreeableness	-.276	.022	-.052	-12.383	< .001	-.319	-.232
Neuroticism	.527	.018	.135	29.502	< .001	.492	.562
***Model summary***							
Variance explained by model	Adjusted R^2^ = .223 (22.3%)						
Statistical significance of model	*F*(8, 50916) = 1826.904, *p* < .001						
**Outcome: Internet Gaming Disorder**							
(Intercept)	20.136	.306	-	65.761	< .001	19.536	20.736
Age	-.002	.003	-.003	-.673	.501	-.009	.004
Gender	.661	.084	.031	7.826	< .001	.496	.827
Weekly time spent gaming	.089	.001	.242	60.923	< .001	.086	.092
Openness	-.149	.045	-.014	-3.349	< .001	-.237	-.062
Conscientiousness	-1.895	.041	-.201	-46.284	< .001	-1.975	-1.815
Extraversion	-.231	.035	-.029	-6.596	< .001	-.300	-.163
Agreeableness	-.587	.043	-.058	-13.762	< .001	-.671	-.504
Neuroticism	1.607	.034	.214	46.948	< .001	1.540	1.674
***Model summary***							
Variance explained by model	Adjusted R^2^ = .226 (22.6%)						
Statistical significance of model	*F*(8, 50916) = 1861.415, *p* < .001						

***Abbreviations***: *B*: unstandardized regression coefficient; SE: standard error; β: standardized regression coefficient; R^2^: R-squared.

Gaming Disorder as measured by the Gaming Disorder Test (GDT); Internet Gaming Disorder as measured by the Internet Gaming Disorder Scale–Short-Form (IGDS9-SF).

For completeness, we also present data informing how many participants met the criteria for disordered gaming according to both frameworks. In the context of the WHO framework, about 2.2% of the total sample met the criteria for GD. In the context of the APA framework, about 5.5% of the sample met the criteria for IGD.

**Fig 1:**
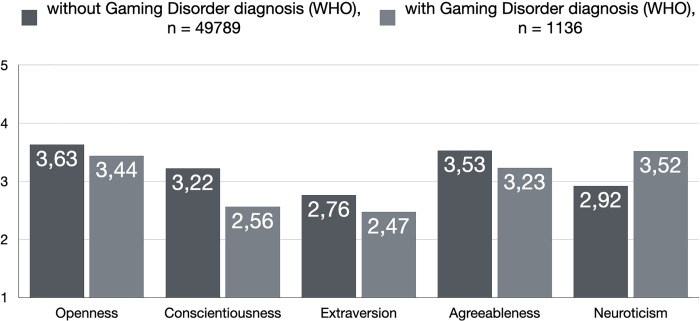
Personality comparisons among potentially disordered and non-disordered gamers according to WHO framework. All contrasts were statistically significant at the *p* < .001 level (SD as follows in the same order as in the figure from left to right; Openness: .58/.66; Conscientiousness: .66/.68; Extraversion: .78/.84; Agreeableness: .61/.68; Neuroticism: 0.83/0.81).

**Fig 2:**
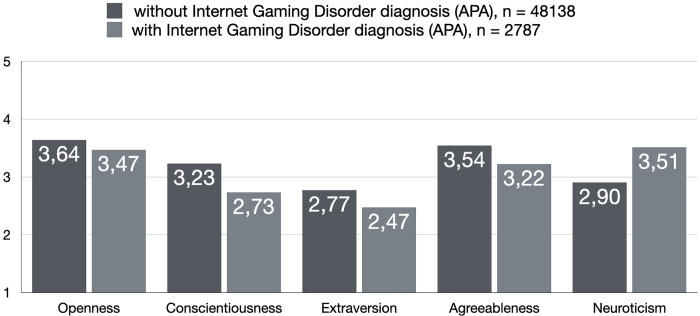
Personality comparisons among potentially disordered and non-disordered gamers according to APA framework. All contrasts were statistically significant at the *p* < .001 level (SD as follows in the same order as in the figure from left to right; Openness: .58/.64; Conscientiousness: .65/.70; Extraversion: .78/.79; Agreeableness: .61/.68; Neuroticism: .82/.79).

To further find support for the personality associations as described in the present text, we also investigated contrasts of potentially disordered gamers against non-disordered gamers across both frameworks. The findings are depicted in Fig 1, Fig 2 and they further support the importance of low conscientiousness and high neuroticism as being the most relevant personality dimensions for understanding disordered gaming. All contrasts were statistically significant at the *p* < .001 level.

## 4. Discussion

The present study sought to explore the interplay between personality and disordered gaming in a multinational and large-scale study using a sample that included participants from 150 countries. This exploratory research investigated whether previously reported associations between personality traits (as captured by the Big Five personality trait model) and disordered gaming would be observed across the APA and WHO frameworks for disordered gaming. Specifically, we aimed at testing the relationship between low conscientiousness and high neuroticism in relation to disordered gaming within a correlational and multiple linear regression predictive modeling approach.

Further, to expand upon recent findings between personality and time spent gaming [19], we explored how time spent gaming and disordered gaming may be differentially associated across the APA and WHO frameworks. To the best of the authors’ knowledge, no previous research has explored the wider scope of potential relationships between personality traits and time spent gaming in the context of the two leading frameworks for disordered gaming (i.e., APA and WHO frameworks).

As observed in earlier works [17, 18], the WHO framework resulted in lower prevalence rates of disordered gaming compared to the APA framework. Furthermore, consistent with the postulated assumptions and previous research, the present findings strengthen the literature by highlighting the links between disordered gaming with low conscientiousness and high neuroticism. Within the APA framework, neuroticism was the strongest “driver” of disordered gaming symptoms while within the WHO framework conscientiousness was the most informative trait (in terms of correlations as shown in Table 2).

The findings obtained in this study reinforce the notion that disordered gaming is a mental health condition that should not be confounded with high gaming engagement (i.e., time spent gaming) [31]. To this end, the results indicated that the magnitude of the encountered associations between gaming behavior and personality traits differed significantly when assessing the relationship between personality and time spent gaming compared to disordered gaming. Since disordered gaming is a mental health condition and a global public health phenomenon [32, 33], the effects stemming from disordered gaming when compared to time spent gaming are expected to be greater in terms of magnitude, which is an important distinguishing feature of this condition since disordered gaming should only be considered in the presence of major functional impairments [34–36]. Interestingly (and as hypothesized), the present findings inform that at the conceptual and clinical levels, personality is generally consistently associated with disordered gaming irrespective of the diagnostic framework utilized in the assessment of this condition.

Against the background of the cultural diversity of the sample and the large sample size investigated, we believe the present study is of universal validity and global significance. This is particularly true for the associations between high neuroticism/low conscientiousness and disordered gaming lying in the moderate effect size area. Please also note that the patterns of high neuroticism/low conscientiousness and higher tendencies towards disordered gaming were robustly visible at the country level (see S1 Data). Furthermore, the association between personality and time spent gaming was significantly lower in terms of magnitude, illustrating that personality is of higher importance regarding disordered gaming associations (see also this work [19]).

## 5. Conclusions

Based on the present findings, this study highlights the role of individual differences in the potential etiology of addictive behaviors, specifically in relation to personality traits as it was found to be significant when understanding its interplay with disordered gaming tendencies. Despite these novel and promising findings, the present study is not without potential limitations. Firstly, the research conducted was limited in nature due to the lack of an experimental or longitudinal design to assess causal links between personality and gaming behaviors as a cross-sectional design was adopted. Secondly, the use of self-report methodologies is arguably problematic when gauging participants’ actual behaviors due to potential self-report biases. Thus, we acknowledge that the present investigation cannot disentangle whether personality causes disordered gaming tendencies or whether it is a consequence of it. Given the stability of personality traits (a simple rule of thumb), one might expect the former pathway to be more likely to occur. However, only future studies using appropriate designs will be able to test this assumption and shed further light on this issue. Moreover, please note that the scoring of the disordered gaming scales was done as described in a recent work [18] and we also note that such scoring needs to be aided by clinical interviews to yield clinically-informed diagnosis (hence this is a limitation of the present approach). Another possible limitation could be inferred from the language in which the survey was administrated. Although non-English speaking participants were excluded from the analyses, possible language barriers could have led to a bias in the findings. The platform used (i.e., www.do-i-play-too-much.com) might also have led to biased findings as it could have attracted gamers who already spend more time on gaming and/or might be more prone to disordered gaming. It is worth noting that the sample was also skewed and presented a relatively low number of female gamers. Finally, several other variables beyond personality traits may be of importance when attempting to understand the etiology of disordered gaming (e.g., life satisfaction, self-esteem, well-being, etc.). Therefore, such further variables should be also investigated in interaction with personality in future research.

Despite these potential limitations, based on the large number of participants in this study, the present insights still might be of merit and relevance for policy makers and stakeholders. Although individuals with low conscientiousness and high neuroticism are slightly more likely to develop higher tendencies towards disordered gaming (hence this is not a perfect association), short screenings of these personality traits might help identifying vulnerable groups, enabling the delivery of preventative measures minimizing harms associated with disordered gaming.

## Supporting information

S1 Data (XLSX)
